# An epizootic of *Chlamydia psittaci* equine reproductive loss associated with suspected spillover from native Australian parrots

**DOI:** 10.1038/s41426-018-0089-y

**Published:** 2018-05-16

**Authors:** Cheryl Jenkins, Martina Jelocnik, Melinda L. Micallef, Francesca Galea, Alyce Taylor–Brown, Daniel R. Bogema, Michael Liu, Brendon O’Rourke, Catherine Chicken, Joan Carrick, Adam Polkinghorne

**Affiliations:** 1 NSW Department of Primary Industries, Elizabeth Macarthur Agricultural Institute, Menangle, NSW Australia; 2 Animal Research Centre, University of the Sunshine Coast, 91 Sippy Downs Drive, Sippy Downs, 4556 QLD Australia; 3 The ithree Institute, University of Technology, Sydney, Ultimo, NSW Australia; 4 Scone Equine Hospital, Scone, 2337 NSW Australia; 5 Equine Specialist Consulting, Scone, 2337 NSW Australia

## Abstract

*Chlamydia psittaci* is an avian pathogen capable of spill-over infections to humans. A parrot *C. psittaci* strain was recently detected in an equine reproductive loss case associated with a subsequent cluster of human *C. psittaci* infections. In this study, we screened for *C. psittaci* in cases of equine reproductive loss reported in regional New South Wales, Australia during the 2016 foaling season. *C. psittaci* specific-PCR screening of foetal and placental tissue samples from cases of equine abortion (*n* = 161) and foals with compromised health status (*n* = 38) revealed *C. psittaci* positivity of 21.1% and 23.7%, respectively. There was a statistically significant geographical clustering of cases ~170 km inland from the mid-coast of NSW (*P* < 0.001). Genomic analysis and molecular typing of *C. psittaci* positive samples from this study and the previous Australian equine index case revealed that the equine strains from different studs in regional NSW were clonal, while the phylogenetic analysis revealed that the *C. psittaci* strains from both Australian equine disease clusters belong to the parrot-associated 6BC clade, again indicative of spill-over of *C. psittaci* infections from native Australian parrots. The results of this work suggest that *C. psittaci* may be a more significant agent of equine reproductive loss than thought. A range of studies are now required to evaluate (a) the exact role that *C. psittaci* plays in equine reproductive loss; (b) the range of potential avian reservoirs and factors influencing infection spill-over; and (c) the risk that these equine infections pose to human health.

## Introduction

*Chlamydia psittaci*, a member of the *Chlamydiaceae* family, is an obligate intracellular pathogen with a broad host range. Birds are the major reservoir for this species with nearly 500 hundred avian species known to be susceptible to infection and disease, the latter commonly referred to as psittacosis^1^. While psittacosis is a concern to animal health, the pathogenic significance of *C. psittaci* is primarily linked to its established role as a globally distributed zoonotic pathogen^1–4^. Inhalation is considered the main mode of pathogen entry with disease severity ranging from a subclinical infection, mild respiratory disease to life-threatening pneumonia and systemic psittacosis. While there have been rare reports of human-to-human transmission of *C. psittaci*^5, 6^, contact with infected birds^7^ or substrates contaminated with bird excreta^3, 8, 9^ appears to be the major route of exposure and potential transmission. Despite its obligate requirement for a host during the replicative phase of its lifecycle, *C. psittaci* elementary bodies are known to persist in soil and water following shedding from infected birds^10^. Aerosolisation of infectious particles from soil has been linked to outbreaks in humans^8, 9^.

*C. psittaci* infections in other mammalian species have been less well-studied with prevalence rates potentially underestimated. *C. psittaci* has been detected in dogs, cats, pigs, cattle, buffalo, goats, sheep and horses^11–15^ in association with respiratory, intestinal and arthritic diseases, as well as reproductive loss. The significance of *C. psittaci* in these diseases has often been unclear due to co-infection with various infectious agents, including other *Chlamydia* spp.^14, 16^. *C. pneumoniae*^17, 18^ as well as *C. abortus* (co-infecting with *C. psittaci*)^11^ were reported in association with respiratory infections in both diseased and healthy horses, whereas *C. abortus* DNA (in co-infection with *C. suis* and *C. psittaci*) was also detected in equine placental samples^19^.

The strongest evidence for the potential of *C. psittaci* to cause infection and disease in a non-human mammalian host has recently re-emerged in horses^20^. *C. psittaci* was previously identified as the most likely cause of reproductive loss in ~14% of horses in a Hungarian study using a combination of immunohistochemical and PCR detection strategies^16^, and was also isolated from an equine abortion case in Germany^21^. In Australia, equine reproductive loss cases have recently come under the spotlight due to a documented zoonotic transmission of *C. psittaci* from equine placental membranes to humans resulting in five cases of psittacosis, a previously unrecognised route of transmission for this bacterium^3, 22, 23^. Multilocus sequence typing (MLST) of the *C. psittaci* strain (Horse_pl) isolated from the placental material of the index case revealed it belongs to the globally distributed, pathogenic avian 6BC-type *C. psittaci* subclade, together with other human and parrot Australian isolates, suggesting a psittacine reservoir for these infections^22^. An avian reservoir was previously suspected based on the identification of *C. psittaci* in association with equine reproductive loss cases in the Hungarian study^16^, but the identity of the avian reservoir and, indeed, the overall prevalence and significance of *C. psittaci* in association with equine reproductive loss remains unknown. To address these questions, we performed a pilot surveillance study of *C. psittaci* infection prevalence in association with equine reproductive loss in a large and intensive thoroughbred horse breeding region of Australia. Unexpectedly, we detected a relatively high levels of *C. psittaci* infection during the sampling period, suggesting that this pathogen may be responsible for a significant number of previously undiagnosed cases of equine reproductive loss. Further, molecular typing and comparative genomics illustrated that the detected strains, again, appear to be of parrot origin, highlighting that native Australian parrots may be a significant reservoir for *C. psittaci* infection spill-over to an unprecedented range of mammalian hosts.

## Results

### Prospective *C. psittaci* screening of equine reproductive loss cases in New South Wales in 2016

The equine pregnancy losses occurred from May through November 2016 and were from 243 to 351 days of gestation. The majority of the foetuses and placentas had changes consistent with acute inflammation. The foetuses had died just before or during delivery and the mares had no signs of systemic infection. The affected newborn foals were less than a week old at presentation and all had severe systemic disease. The mortality rate in newborn foals was very high and death occurred rapidly after the development of clinical signs.

Of the 161 equine abortion cases examined, 34 tested positive for *C. psittaci* in real-time PCR, giving a prevalence of 21.1%. Of the 38 cases in which foals were carried to term but were of compromised health status at parturition, 9 (23.7%) tested *C. psittaci* positive. Seven of the nine compromised foals positive for *C. psittaci* later died. The prevalence of *C. psittaci* infection detected in this study across all cases on all properties was 43/199 (21.6%). Equine herpesvirus-1 (EHV-1) infection was detected at much lower prevalence, with only 9 of the 199 cases testing positive (4.5% prevalence). One case was positive for both EHV-1 and *C. psittaci*. This was a compromised newborn foal that died 24 h after admission to the intensive care unit. The gross and histopathology indicated that the cause of the perinatal death was EHV infection. The histopathology of foetal and placenta tissues from 134 cases of equine abortion or neonatal death was examined by two veterinary pathologists who were unaware of the *C. psittaci* status of the cases. Of these 134 cases, 30 were positive for *C. psittaci*. The histopathology of 28 of the 30 positive cases was consistently described as acute non suppurative interstitial pneumonia, vasculitis, hepatitis, deep chorionitis and allantoitis, amnionitis and funisitis. One case had histopathology consistent with EHV infection (described above) and the other case had mild neutrophillic amnionitis and funisitis. Bacteria were isolated in only 3 of the 30 *C. psittaci*-positive cases. Placentitis caused the reproductive loss in 53% of the 134 cases with *C. psittaci* identified as the most likely cause of placentitis (43%). Other causes of placentitis included Equine Amnionitis and Foetal Loss (EAFL) (17%), suspect EAFL (11%), ascending placentitis (14%), placentitis of unknown origin (11%) and focal mucoid placentitis (4%). The remaining causes of pregnancy loss and perinatal death included poor blood flow (21%), congenital abnormalities (4%), EHV (3%), hypoxic perinatal death (5%), perinatal infection (1%) and unknown diagnosis (13%).

**Fig. 1:**
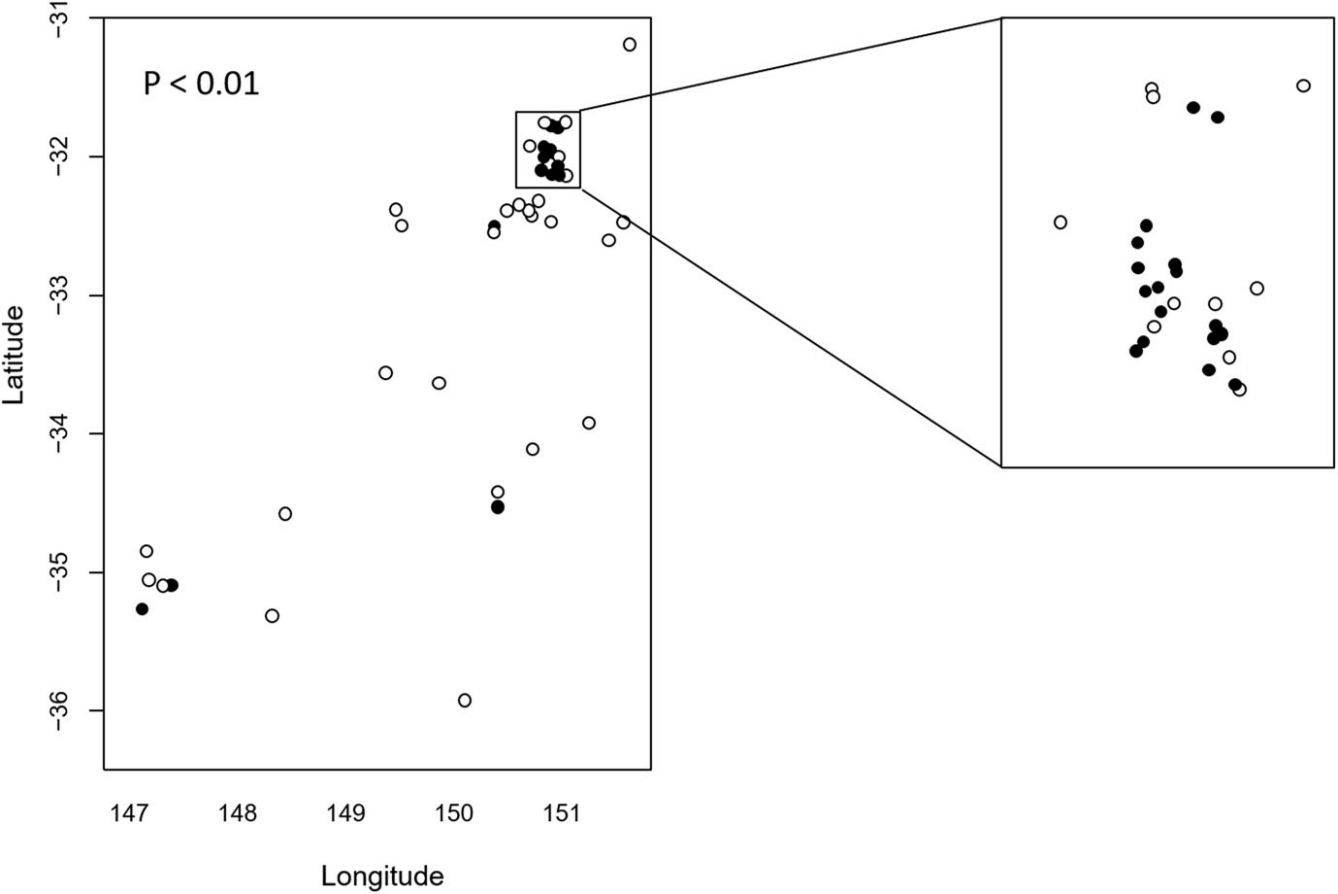
Statistical analyses of case clustering. The proportion of properties with *C. psittaci* positive cases within and outside the boxed area was examined using a comparison of proportions. There was statistically significant clustering of *C. psittaci* cases within the boxed area (*P* < 0.001)

*C. psittaci* positive cases were detected at 21 out of 54 sampled properties and were clustered ~170 km inland from the NSW mid-coast (Figure S1). While there were insufficient data to fit a smooth spatial trend across the entire survey area to determine probability of positivity across all locations, we compared proportions of properties with cases positive for *C. psittaci* within (*n* = 27) and outside (*n* = 27) of the NSW mid-coast area [Lat −31.7 to −32.1; Long 150.7 to 151.1] (boxed, Fig. 1). We also compared the proportion of total positive cases within and outside that area. The proportion of properties with positive cases was significantly higher within (17/27; 63%) compared to outside (4/27; 14.8%) the boxed area (*P* < 0.001). There were also a significantly higher proportion of total *C. psittaci* positive cases within (37/128; 28.9%) compared to outside (6/71; 8.45%) the boxed area (*P* < 0.001). These data are suggestive of a disease cluster around Lat −31.7 to −32.1; Long 150.7 to 151.1. Interestingly, two of the four positive properties outside the mid-coast area were located at Wagga Wagga, the site of the index case and further subsequent positive detections in 2017 (unpublished data), suggesting that Wagga Wagga may represent a second disease cluster.

**Fig. 2:**
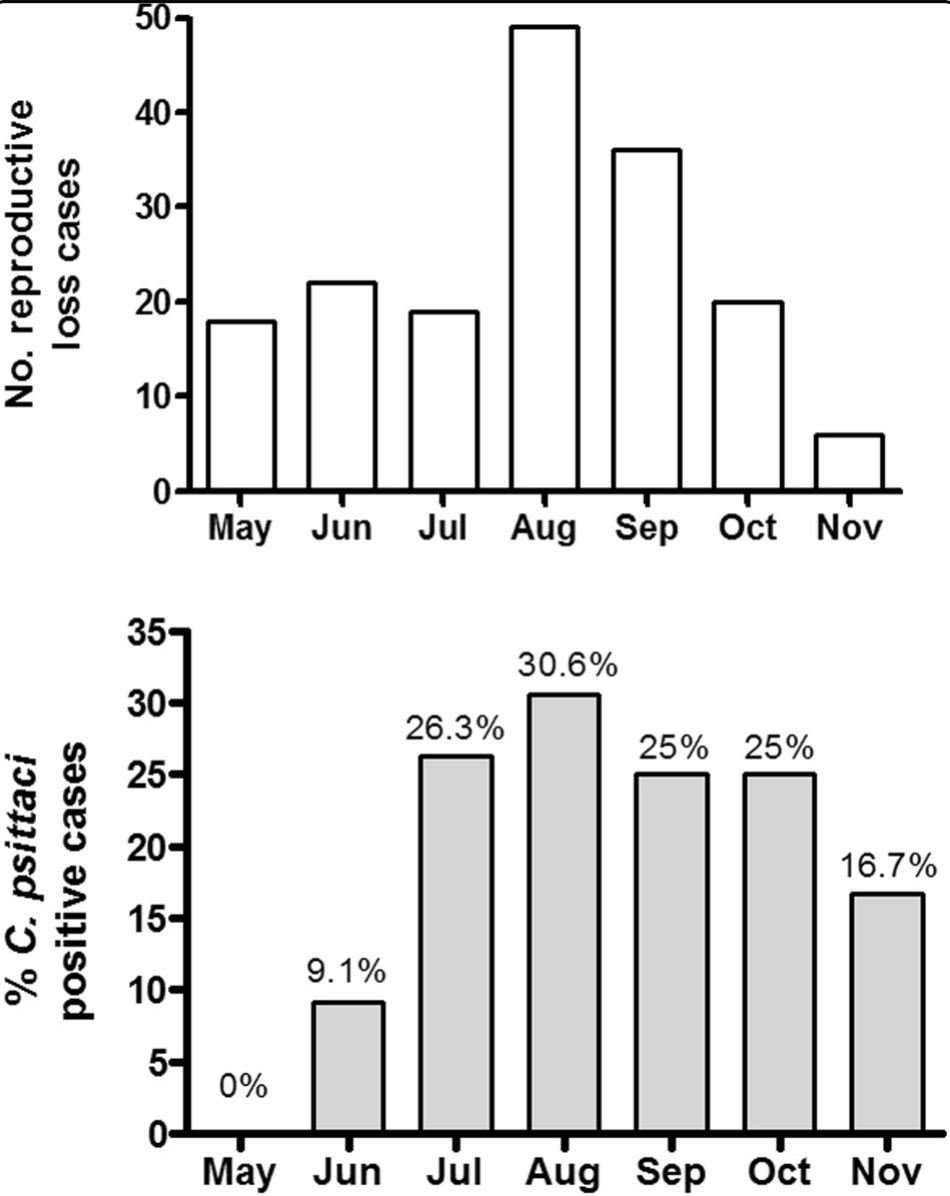
Rates of equine reproductive loss relative to *C. psittaci*positivity in the 2016 foaling season. Temporal distribution of equine reproductive loss cases (**a**) and the proportion of *C. psittaci* positive cases (**b**)  are shown.

We examined the temporal distribution of cases of equine reproductive loss across the 2016 foaling season (Fig. 2a) and compared to the proportion of *C. psittaci* positive cases detected (Fig. 2b). There was a large increase in the proportion of *C. psittaci* cases detected in July compared to May and June. The proportion of positive cases detected peaked in August but remained high from July through to October, eventually declining in November.

Of the cases for which paired placental-foetal samples were available (*n* = 76), there was a high level of concordance (95%) between results for each tissue type. Of the 76 cases, 25 were positive for *C. psittaci* in both foetal and placental samples, while 49 tested negative for *C. psittaci* in both samples. One sample tested negative in placental but positive in foetal tissue and a further one sample tested negative in the foetal but positive in the placental tissue (Table S1). A subset of 20 *C. psittaci* negative foetal and/or placental samples were also tested with a pan-*Chlamydiales* assay in a separate study^24^ and were found to be negative for other members of this Order.

Surveillance was also undertaken on mares across eleven properties which had reported equine abortions. Tested mares were either cohorts of aborting mares or mares with a prior history of aborting foals or delivering foals with compromised health. Placental and/or foetal samples collected from abortion cases on 9 of the 11 properties had tested positive for *C. psittaci*, while samples collected from the remaining properties were negative. *C. psittaci* was detected in only a single-vaginal swab from a mare that had aborted in the days prior to sampling. Follow-up testing of the same mare 12 days later returned a negative result for *C. psittaci*.

### Investigation of *C. psittaci* load and distribution in equine reproductive material

**Fig. 3:**
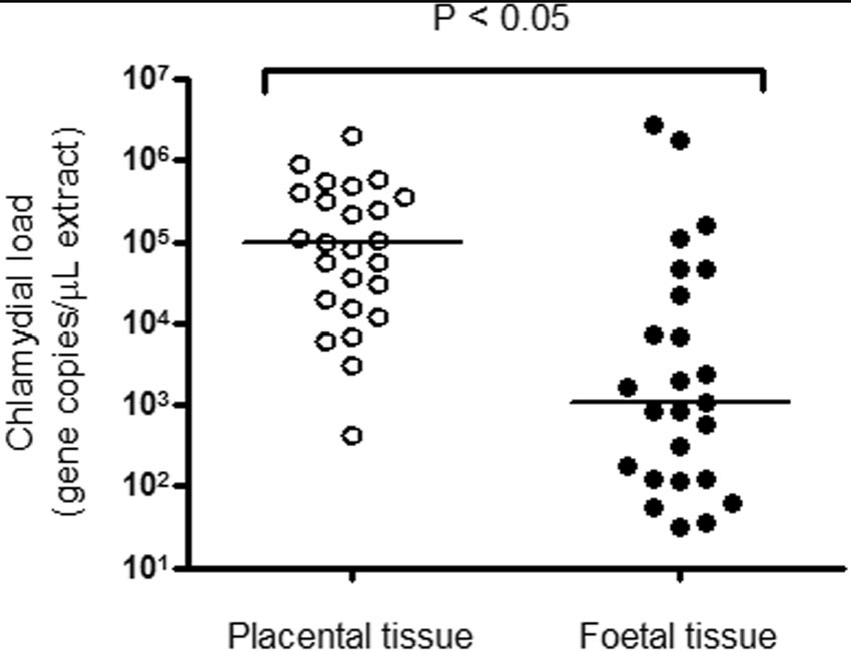
Analysis of *C. psittaci* load in equine placental and foetal tissue. Median chlamydial load (bars) in paired placental (open circles) and foetal tissues (closed circles) are shown and are statistically different.

qPCR was used to determine loads of *C. psittaci* in the 25 paired positive placental and foetal tissues, as well as the placental material linked to five zoonotic psittacosis cases^3^. The mean, median and range of *C. psittaci* gene copy numbers per µL of DNA extract from the paired tissues are shown in Table S2. The median load of *C. psittaci* was significantly higher in placental tissue using a two-tailed Mann–Whitney test (Fig. 3; *P* < 0.05). Median chlamydial load in foetal tissues was ~2 orders of magnitude lower than observed in placental tissues. No significant correlation was observed between chlamydial load in placental versus foetal tissues (*r* = 0.13; *P* > 0.05).

**Fig. 4:**
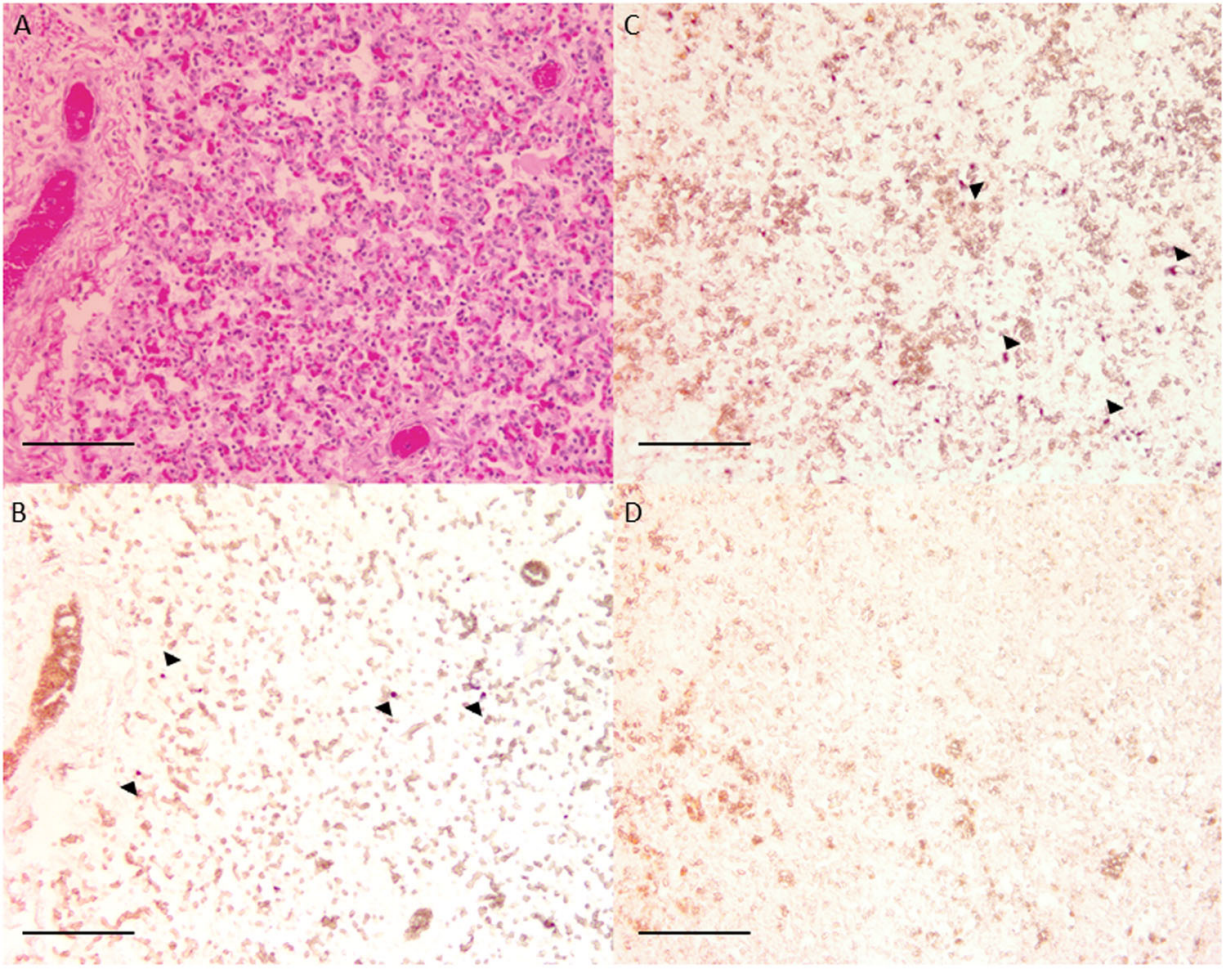
In situ hybridisation of equine foetal tissue. Equine lung tissue stained with haemotoxylin and eosin (**a**) and a *C. psittaci* probe (**b**) revealed focal intracellular accumulations of probe similar to *C. psittaci*-infected avian kidney tissue (**c**), where arrows indicate the accumulations of the probes. Staining was not observed in the absence of *C. psittaci*-specific probe (**d**)

In situ hybridisation was used to confirm the presence of *C. psittaci* within equine foetal tissues (Fig. 4a–d). Unfortunately, no placental tissue samples were available for this method. Serially sectioned equine lung tissue stained with haematoxylin and eosin (Fig. 4a) and a *C. psittaci*-specific probe (Fig. 4b) revealed focal areas of intracellular staining similar to infected avian tissue (Fig. 4c). Cells staining positive for *C. psittaci* appear to be foetal monocytes; however, a more detailed description of the pathology is in preparation.

### Whole-genome sequencing of equine epizootic *C. psittaci* strains

Full *C. psittaci* genome sequences with almost 100% chromosome coverage were successfully obtained from five clinical samples: the placental isolate from the Wagga Wagga index case (Horse_pl), and four samples from the inland mid-coast cluster (paired samples 8882_placenta and foetus, 9945_foetus and 10652_placenta) (Table 1). Whole-genome alignment of the Australian horse *C. psittaci* strains from this study and previously described *C. psittaci* from various hosts confirmed high sequence conservation and synteny characteristic for chlamydial species^25^. Newly sequenced strains had an approximate 1.15 Mbp chromosome size with an average of 1000 predicted CDS and a 39.05% GC content. All isolates also carry the characteristic 7.5 kbp chlamydial plasmid containing eight CDS. The plasmid sequences had 100% sequence similarity (Table 1). The primary contaminating DNA in the sequenced samples was that of a horse (*Equus caballus*) as determined by BLAST analyses performed in this study (data not shown).

**Table 1:** Description of equine *C. psittaci* strains and their genomes sequenced in this study

	**Horse_pl**	**8882_placenta**	**8882_foetus**	**9945_foetus**	**10652_placenta**	**9945_placenta**	**CR394**
Host and clinical presentation	Equine, placentitis	Equine, placentitis	Equine, placentitis	Equine, placentitis	Equine, placentitis	Equine, placentitis	Parrot, psittacosis
Sample type	Placenta tissue DNA extract	Placenta tissue DNA extract	Foetal tissue DNA extract	Foetal swab DNA extract	Placental swab DNA extract	Placental swab DNA extract	Cultured isolate
Region	Wagga Wagga	Inland mid-coast	Inland mid-coast	Inland mid-coast	Inland mid-coast	Inland mid-coast	Blue mountains
Total no. of filtered reads	1,639,666	53,441,316	55,654,176	55,153,664	52,625,864	53,199,404	49,658,992
Avg. read length	250 bp	125 bp	125 bp	125 bp	125 bp	125 bp	125 bp
No. of contigs	1 (1.169 Mbp)	16 (2.4–200 kbp)	17 (1–288 kbp)	7 (2.8–308 kbp)	8 (2.3–323 kbp)	1	1
Avg. read coverage for de novo	32.89×	256.11×	16.25×	181.95×	319.22×	32.65×	14×
Draft genome size (Mbp)	1.169	1.157	1.163	1.167	1.162	0.127	0.127
% GC	39.05	39.05	39.05	39.05	39.05	39.05	39.05
No. of predicted CDS	1030 (319 hp*)	1017 (310 hp)	1016 (310 hp)	1013 (306 hp)	1017 (310 hp)	—	—
Plasmid size	Yes (7.5 kbp)	Yes (7.5 kbp)	Yes (7.5 kbp)	Yes (7.5 kbp)	Yes (7.5 kbp)	Yes (7.5 kbp)	Yes (7.5 kbp)
No. of mapped reads (proportion of filtered reads)	185,824 (11.3%)	2,452,352 (4.59%)	159,398 (0.29%)	2,238,890 (4.06%)	2,818,547 (5.36%)	57,114 (0.11%)	275,490 (0.55%)

The remaining equine (9945_placenta) and parrot (CR394) *C. psittaci* genomes were poorly assembled with either low-read depth or poor chromosome coverage, hence were omitted from further comparative genomics. However, using read mapping to the 217 kbp core conserved genome contig, we were able to successfully assemble core genome contigs of the paired samples for 9945 (placenta), as well as a *C. psittaci* CR394 isolated from Crimson Rosella (*Platycercus elegans*) from the Blue Mountains, NSW^2^. The 271 kbp contigs had 32.7× coverage for 9945_placenta, and 14× coverage for CR394 strain (Table 1). The *C. psittaci* whole as well as core genomes from the mare rectal swab and parrot tissues samples were very fragmented with a read depth of <10×, hence these genomes were deemed unusable for further analyses.

### Core genome phylogeny reveals that the Australian equine *C. psittaci* strains are clonal and likely of avian origin

**Fig. 5:**
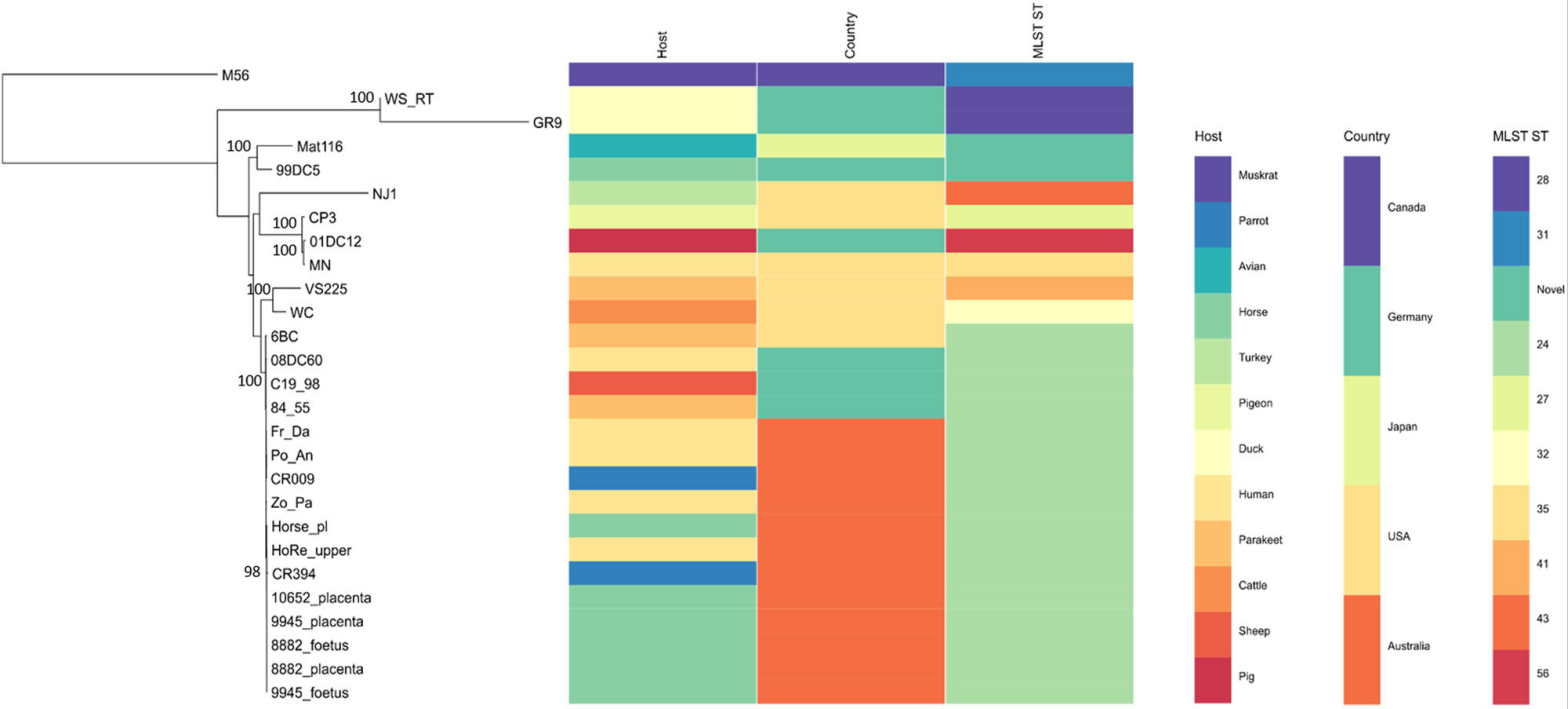
Phylogeny of the newly sequenced Australian equine epizootic *C. psittaci* strains. Mid-point rooted RAxML phylogenetic tree with 500 bootstraps was constructed using 217 kbp core conserved genome alignment of the 27 *C. psittaci* from various hosts. Bootstrap support is displayed on the tree nodes. Host, country and MLST for each strain is included on the right. Image was drawn using Phandango

To assess the diversity of the equine epizootic strains circulating in NSW within the context of other global and Australian animal and human *C. psittaci* strains, we constructed a mid-point rooted RaXML phylogenetic tree using a 271 kbp conserved core genome alignment of a total of 27 strains from avian, livestock, human and other mammalian hosts (Fig. 5). Phylogenetic analyses from this study revealed that all Australian equine strains cluster tightly within the globally disseminated, pathogenic and clonal avian 6BC/ST24 type clade^26^, which also includes other previously described Australian parrot and human isolates^2^. Besides the clonal 6BC clade, the phylogenetic analyses in our study resolved additional seven distinct clades, with similar clustering of the isolates as previously assessed using these global *C. psittaci* isolates^2, 26^.

**Fig. 6:**
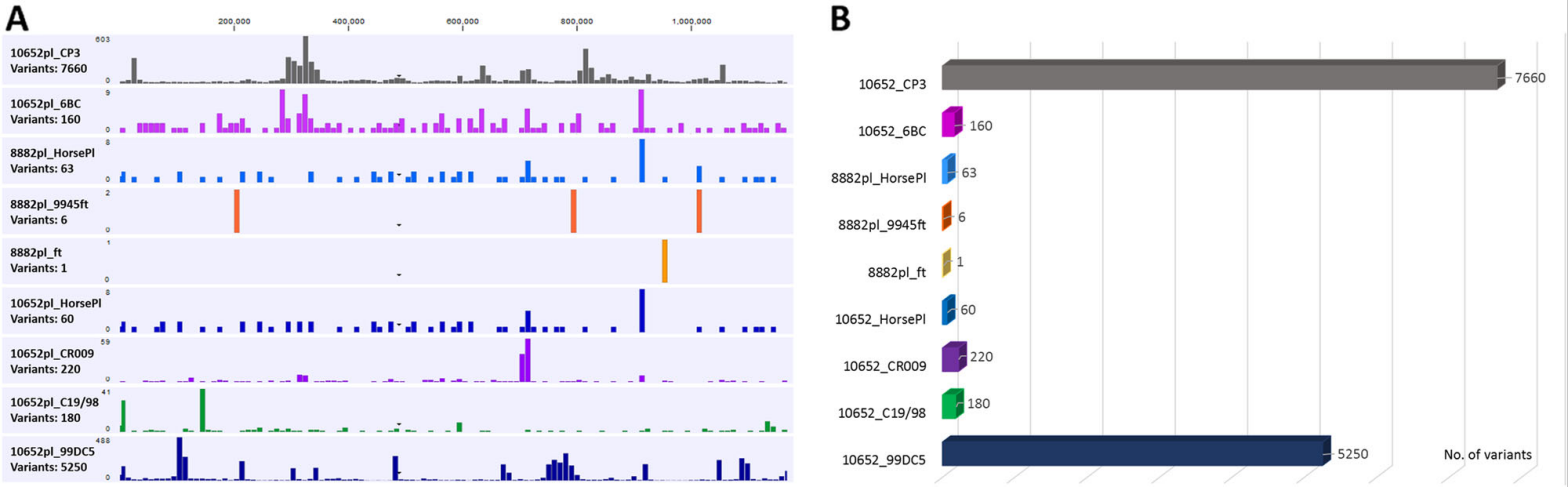
Genetic diversity of the Australian equine epizootic *C. psittaci* strains. **a** Distribution of polymorphisms (variants including SNPs and indels) across the chromosome of the equine strains from this study in comparison to distantly, as well as closely related strains. No. of variants (*y*-axis) are represented as histograms in a 10 kbp chromosomal region (*x*-axis), and were determined by read mapping of the query strain to the nominated reference strain genome (e.g., 10652pl reads mapped to CP3 genome; 8882pl reads mapped to HorsePl genome). **b** Total number of variants for each read mapping represented in a line graph

Comparative genomics of the full-length equine *C. psittaci* draft genomes of samples from the mid-NSW coast region further confirmed the phylogeny and clonality of *C. psittaci* of those cases. The strains possess less than five SNPs between them, leading to the presumption that a single clonal *C. psittaci* strain is responsible for this documented epizootic (Fig. 6). In comparison to the Horse-pl strain from the Wagga Wagga index case, the mid-NSW coastal strains differed by up to 63 SNPs, evenly distributed around the chromosome. A similar degree of variation with less than 100 SNPs was also observed when comparing Australian equine *C. psittaci* strains to the previously sequenced Australian parrot and human isolates, indicating that the Australian strains are very closely related (Fig. 6). The Australian equine strains differed by ~200 SNPs from the parrot 6BC, sheep C19/98, and human 08DC60 strains, confirming previously observed shared ancestral origin to the 6BC/ST24 clade.

Australian equine strains differed by average of 7650 SNPs to more distantly related pig, pigeon and human *C. psittaci* isolates (01DC12, CP3 and MN, respectively). In comparison to the clade consisting of the German mare 99DC5 isolate, our equine strains differed by an average of 5200 SNPs. When compared to these isolates, higher density of SNPs was observed in the chlamydial polymorphic membrane protein (*pmp*’s) coding regions (Fig. 6).

### Molecular typing of the Australian epizootic equine *C. psittaci* strains confirms 6BC-type genotype

To further evaluate the genetic diversity of the equine epizootic strains circulating in NSW, we applied MLST on a total of 22 *C. psittaci* PCR positive samples from the mid-coastal NSW epizootic (including the whole-genome sequenced strains), and compared these STs to additional previously typed global and Australian *C. psittaci* strains (Table S3).

**Fig. 7:**
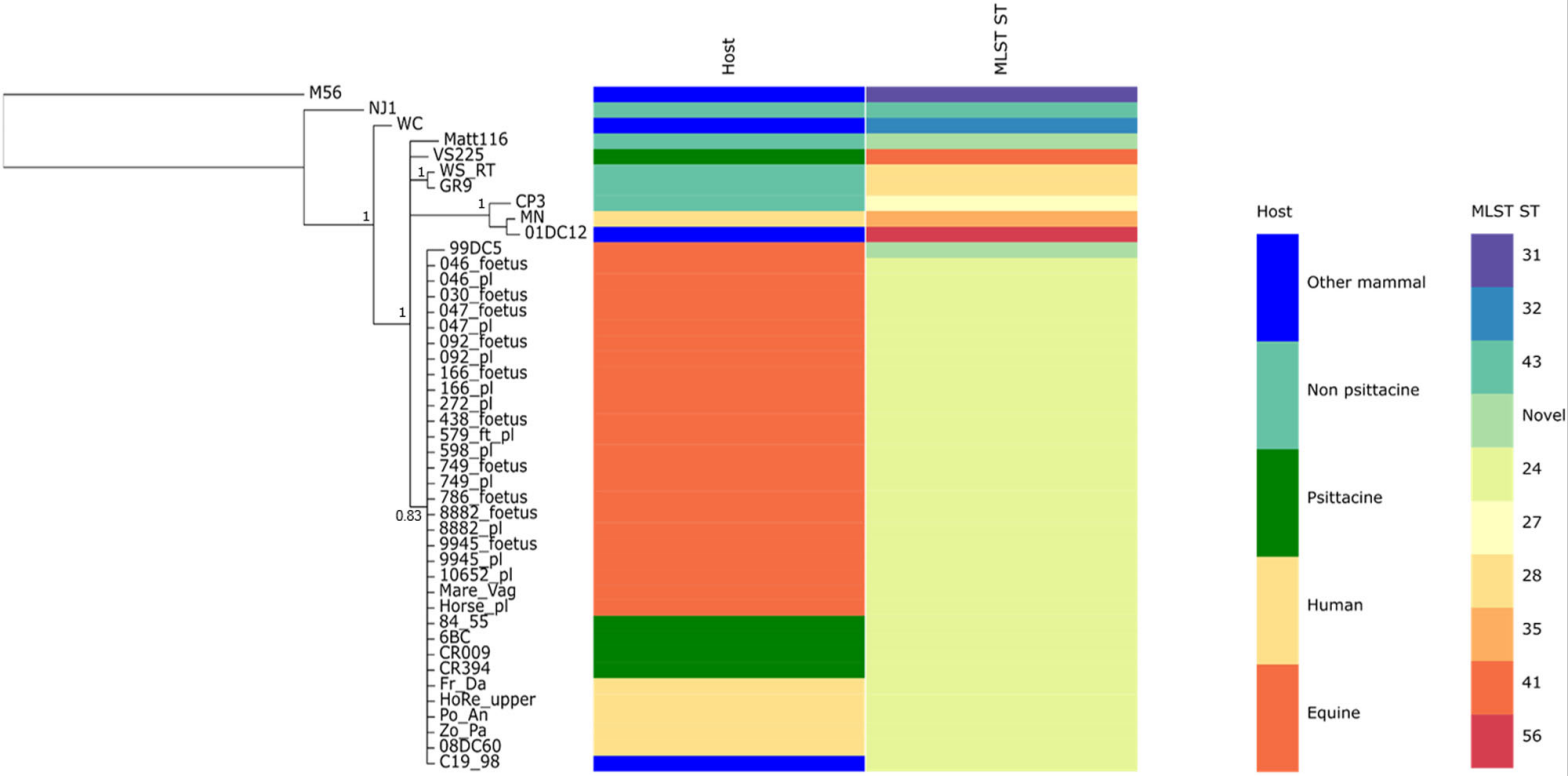
MLST derived Bayesian phylogeny of the Australian equine epizootic *C. psittaci*. A mid-point rooted Bayesian phylogenetic tree was constructed using the alignment of the concatenated MLST fragment sequences from 44 *C. psittaci* strains from various hosts. Posterior probabilities are displayed on the nodes. Hosts and STs are represented by various colours as indicated in the legend. Image was drawn using Phandango

As observed in the Bayesian phylogenetic tree constructed from the alignment of concatenated MLST fragments (Fig. 7), all equine strains share the same ST (ST24), the same ST shared by the previously typed Horse_pl^22^, Australian parrot and human isolates from the Blue Mountains outbreak^2^.

## Discussion

Among members of the genus *Chlamydia*, *C*. *psittaci* appears to have the most cosmopolitan distribution, infecting a wide range of hosts and tissue types and causing asymptomatic to fulminant disease episodes^1, 25^. While the host range continues to expand for this pathogen, avian hosts nevertheless appear to be the common denominator in the epidemiology of this pathogen. In our recent molecular and epidemiological investigations of the first Australian reported case of *C. psittaci* equine reproductive loss associated with a subsequent zoonotic event^3, 22, 23^, we found that the *C. psittaci* strain associated with this case belonged to an evolutionary lineage of this pathogen found in parrots, leading to the suggestion that spill-over from Australian parrots was responsible for this equine infection. In the critical absence of other information about the prevalence of *C. psittaci* infection in equine reproductive loss, we performed a surveillance study of reproductive loss cases from horses in regional NSW.

The results of this work revealed that *C. psittaci* infection was present in tissues associated with equine reproductive loss at a relatively high (21.6%) prevalence. In a previous study from Germany, *C. abortus* and *C. suis* were detected by qPCR in horse placental samples^19^; however, when a subset of 20 *C. psittaci* negative samples from the equine reproductive loss cases in this study was screened for the presence of other *Chlamydia* spp., no positives were detected^24^. Perhaps, this is not surprising as *C. abortus* is currently considered exotic to Australia^27^ and there have been no reports of other “zoonotic” chlamydial species such as *C. suis* and *C. pneumoniae* in equine hosts in Australia. The role of *C. psittaci* in equine abortion has been poorly studied globally despite a small number of studies suggesting an infection prevalence of between 20 and 83% in foetal or placental tissues in parts of Europe^16, 28^. An investigation of equine reproductive loss in Hungary represents the most thorough study of the role of *C. psittaci* in abortion to date and demonstrated an infection prevalence of 83% using PCR and immunohistochemical techniques. In that study, *C. psittaci* could be clearly associated with abortion in only 14.3% of cases, with non-infectious causes (e.g., trauma, umbilical cord torsion, congenital defects) and other infectious agents such as viruses accounting for 20 and 30% of cases, respectively^16^. In contrast, equine herpesvirus accounted for only a small proportion of cases (4.5%) in this study and thorough investigation of 134 of the losses in this study indicated that 22% could be clearly associated with *C. psittaci* infection. When *C. psittaci* was detected, it was considered the cause of the abortion or neonatal death in 28 of 30 (93%) of cases. In contrast to the Hungarian study, *C. psittaci* was detected in only 2 cases where an alternate diagnosis was made. In addition, *C. psittac*i was not detected in any case where there was no inflammation of the foetus or foetal membranes. The relatively high prevalence of *C. psittaci* infection during 2016 maybe unusual and continued monitoring of equine reproductive losses is required to determine the true prevalence of the infection. The detection of *C. psittaci* in association with an equine abortion case in Wagga Wagga in 2015^22^ combined with the prevalence data presented in this study, suggest that *C. psittaci* may be an underdiagnosed cause of equine reproductive loss. Indeed, investigation into this issue was motivated by the zoonotic cases reported in 2015^3^.

The loads of *C. psittaci* detected in placental and foetal tissues varied between cases, but were sufficiently high to induce suspicion that this organism was directly associated with equine reproductive loss. Furthermore, the fact that *C. psittaci* loads were statistically higher in the placenta, combined with gross observations of placentitis, suggests damage to the equine placenta by this pathogen as a precipitating factor in these equine abortion cases. Notably, mild lympho-histiocytic placentitis was the only pathological change detected in *C. psittaci*-infected horses in Hungary^16^. While there was no significant correlation between loads of *C. psittaci* in placental versus foetal tissues, high loads of *C. psittaci* (>1 × 10^6^ gene copies/µL of DNA extract) were sometimes observed in foetal tissues (Fig. 4). Interestingly Szeredi et al.^16^ were unable to detect *C. psittaci* in foetal tissues using either PCR or immunohistochemistry, despite a high prevalence of the pathogen in placental tissues. In this study, we were also able to confirm the presence of *C. psittaci* in what appeared to be foetal monocytes in the foetal lung.

Using comparative genomics analysis of *C. psittaci* strains from the equine chlamydiosis epizootic, we revealed that the strains from geographically distinct studs were identical. Although indicative of horse to horse transmission, the detection of identical strains from these equine reproductive loss cases could not be explained by movement of horses (data not shown). Instead, our demonstration that the strains again clustered closely to strains in the globally distributed, pathogenic and clonal 6BC/ST24 *C. psittaci* clade points to a common Australian parrot reservoir on the basis that (i) 6BC/ST24 strains are primarily reported in parrots, whereas the non-psittacine avian hosts are typically infected with genetically distinct *C. psittaci* lineages^29^; and (ii) our own molecular typing studies of isolated Australian parrot strains showed that these also belong to the 6BC/ST24 lineage^2, 22^. Unfortunately, in terms of the latter, precious little published data are available on the genotype of *C. psittaci* strains predominately found in Australian parrots despite the fact that psittacosis has been recognised as an endemic disease of Australian parrots since at least 1935^30^. If parrots are indeed involved, the exact species of parrot involved in this epizootic is currently unknown with the inland mid-coast area home to a range of endemic parrot species. If parrot spill over is responsible for transmission to horses, we can only hypothesise that, similar to our recent report of a human psittacosis cluster, environmental contamination with *C. psittaci* infected parrot excreta might be sufficient for exposure and subsequent colonisation of pregnant mares. The temporal distribution of cases examined in this study suggests that seasonal and/or environmental factors may contribute to infection prevalence.

While our genetic evidence points primarily towards a role of psittacine 6BC/ST24 strains in Australian equine reproductive loss cases, the fact that (i) equine reproductive loss cases have been reported in other parts of the world where parrots are not endemic^16^; and (ii) the equine strains sequenced in this study are genetically different to the other equine *C. psittaci* strains, such as the previously sequenced *C. psittaci* from a horse in Germany (GenBank accession number KE356169) and the recently described *C. psittaci* from an equine abortion case from Queensland region in Australia^31^, suggests that other *C. psittaci* evolutionary lineages may also have the potential to infect horses as well. Additional work is required to confirm the reservoirs of these equine *C. psittaci* infections and the host, pathogen and environmental factors that might influence these events at the domesticated animal/wildlife interface.

The fact that the index case that prompted this surveillance involved zoonotic transmission to humans means that the detection of an unexpectedly high number of *C. psittaci* positive cases in horses is also of relevance to public health surveillance of human psittacosis cases. While not documented in this current investigation, at least three suspected cases of human psittacosis were recorded in this region which were associated with equine positive cases (unpublished data), further highlighting the potential implications of this work to public health surveillance. The relative risk of exposure to *C. psittaci* infected horses to human health from these equine infections remains unclear with a study to actively follow-up human contacts from other equine cases described in this study failing to detect any acute psittacosis despite intensive surveillance effort^23^. On this basis, we hypothesise that zoonoses from horses are relatively rare and probably affected by (i) the level of intimate contact and/or aerosolisation of *C. psittaci* involved in the human contact with infected tissue; and (ii) the relative level of *C. psittaci* present in the infected material, with our current study showing an extensive range of *C. psittaci* loads in foetal and/or placental tissues. The exact risk obviously requires further detailed investigation. Answers to these questions, as well as the importance of these infections to animal health will only come through detailed ‘One Health’ collaboration between scientists, veterinarians and human health clinicians both here in Australia and abroad.

## Materials and methods

### Case details and sampling performed

Throughout 2016, following the initial index case of *C. psittaci*-associated placentitis^3, 22^, placental and foetal tissues and/or tissue swabs were collected from a total of 199 cases of equine pregnancy loss (*n* = 161) or compromised newborn foals (*n* = 38) from 54 properties in New South Wales, Australia (Figure S1). Cases examined per stud ranged from *n* = 1 to *n* = 22. A postmortem examination was performed on 134 of the cases by experienced equine clinicians and standard samples of lung, liver, thymus, spleen, chorioallantois, amnion and umbilical cord were collected for histopathological examination. The tissues were submitted to a commercial veterinary pathology laboratory for histopathological examination of the tissues by experienced veterinary pathologists (Vetnostics, North Ryde, NSW 2113). Samples of lung aspirate and stomach contents were cultured aerobically for 48 h.

Swabs of placental and pooled foetal tissues (lung, liver, thymus and spleen) were collected from abortion cases, while rectal and nasal swabs were collected from newborn foals with compromised health status. Tissues were collected from newborn foals at postmortem in cases where they subsequently died. All swabs were suspended in phosphate buffered saline containing 0.1% gelatin (PBSG) for transport and storage at −80 °C prior to diagnostic testing. Paired placental and foetal samples were available for 76 of the 199 cases. Paired placental (*n* = 27) and foetal tissue (*n* = 27) samples from positive cases were stored at −80 °C for quantification of chlamydial load. Frozen archived tissues from the 2014 chlamydial equine reproductive loss index case^3, 22^ were also used in this study for comparative purposes.

Surveillance for *C. psittaci* was also carried out on mares across 11 of the 54 properties from which equine abortions were reported in 2016. Mares (*n* = 53) included those that had previously aborted or produced foals of compromised health status (*n* = 13) and cohorts of currently aborting mares (*n* = 40). One mare that had aborted previously was sampled on two occasions 12 days apart. Vaginal swabs (*n* = 54) were collected into PBSG and stored at −80 °C also for diagnostic testing.

### DNA extraction

DNA was extracted from 100 µL of PBSG (swabs) or 20 mg of tissue, using the DNeasy Blood and Tissue kit (Qiagen, Alameda, California) according to the manufacturer’s instructions. DNA was eluted in 100 µL of molecular grade water and stored at −20 °C until required.

### Quantitative PCR (qPCR) screening

qPCR was performed on extracted DNA using the CPS100 and CPS101 primers targeting the 16S rRNA gene/16S–23S rRNA spacer^32^. Reactions contained 10 µL of 2× SensiMix SYBR Lo-ROX, 0.25 µM of each primer and 1 µL of template DNA in a total volume of 20 µL. Cycling was carried out in an AB7500 thermal cycler with an initial 10 min denaturation at 95 °C, followed by 35 cycles of denaturation at 94 °C for 30 s, annealing at 53 °C for 30 s and extension at 65 °C for 30 s and one cycle of 58–72 °C for 5 min. Samples with melt curves within the range of 81.5 °C ± 0.3 °C were considered positive for *C. psittaci*. All samples were simultaneously tested for equine herpesvirus-1 in the Elizabeth Macarthur Agricultural Institute Virology Laboratory using the method of Diallo et al.^33^. A subset of 30 placental and/or foetal DNA samples was also previously screened for presence of other *Chlamydia* spp.^24^.

### Quantitation of chlamydial loads from equine tissues

To determine chlamydial loads in infected samples, a total of 46 samples consisting of 23 pairs of pooled foetal tissues and corresponding placental tissues from *C. psittaci*-positive equine abortion cases were examined. Quantitation was performed using the previously described qPCR assay targeting the *omp*A gene^19^, with the exception that reactions were carried out using TaqMan Environmental Master Mix (Applied Biosystems, Foster City, California, USA). A plasmid standard was used to estimate gene copy number and consisted of the *C. psittaci omp*A gene inserted into the pET21a+ vector (GenScript, Nanjing, China). A dilution series of the plasmid corresponding to 10^−1^ up to 10^7^ gene copies was made in transfer RNA (Sigma-Aldrich, St Louis, USA) and was used to calculate the chlamydial *omp*A gene copy number in each sample.

### In situ hybridisation

For in situ hybridization, a DIG-labelled probe targeting the 16S rRNA gene/16S–23S rRNA spacer was generated using the previously described *C. psittaci* primers (22). The probe was labelled using the PCR DIG probe synthesis kit (Roche, Basel Switzerland) according to the manufacturer’s instructions. Tissues fixed in neutral buffered formalin were paraffin-embedded and 5 µm sections were placed onto Superfrost Plus slides (Menzel Gläser, Thermo Fisher Scientific, Massachussetts, USA). Sections were dewaxed in xylene and rehydrated in an ethanol series. Sections were treated with Proteinase K (Dako/Agilent, Santa Clara, USA) overlaid with a hybridisation coverslip for 15 min in humid chamber 37 °C. Sections were then washed with Tris buffer (0.1 M, pH 8.0) for 3 min at RT and prehybridised for 1 h with 200 µL of prehybridsation solution (50% formamide, 4× saline sodium citrate (SSC) buffer, 1× Denhardt’s solution, 0.25 mg/mL yeast tRNA, 10% dextran sulfate) in a humid chamber at 37 °C. For positive control and test slides prehybridisation solution was exchanged for hybridisation solution which contained 5 ng/µL probe. Negative control slides received no probe. Slides were covered with a hybridisation coverslip and heated to 95 °C for 5 min in a humid chamber. Slides were placed immediately on ice and then transferred to a 42 °C humid chamber and incubated overnight. The next day, slides were washed in washing buffer (Roche) at 40 °C for 10 min. Sections were blocked with 500 µL blocking buffer (Roche) at RT for 30 min. Blocking buffer was then exchanged for a 1:200 solution of anti-DIG antibody diluted in blocking buffer and the slides incubated at RT for 1 h. Excess antibody was removed with for 30 min in wash buffer, slides were equilibrated for 5 min in detection buffer (Roche) and incubated with 500 µL of NBT/BCIP chromogenic solution (Sigma-Aldrich) for 4 h. Slides were rinsed with water, air dried and mounted in aqueous mounting medium.

### Statistical analyses

The significance of geographical clustering of properties with *C. psittaci* positive cases inland from the NSW mid-coast (coordinates 32°00S; 150°50E) was tested using a comparison of proportions in the program R^34^. The median chlamydial load in each tissue type was compared using a two-tailed Mann–Whitney test in GraphPad Prism v4.02. A two-tailed test of Spearman’s rank correlation was used to determine whether there was a significant correlation between chlamydial load in foetal and placental tissues.

### Targeted sample enrichment

For whole-genome sequencing, a total of nine DNA extracts from seven equine placental and foetal samples from the epizootic, a parrot tissue sample from central NSW and *C. psittaci* CR394 isolate from a Crimson Rosella parrot from Blue Mountains endemic region were treated with the NEBNext Microbiome DNA Enrichment kit (New England Biolabs, Ipswich, Massachussets, USA) to deplete host methylated-DNA, followed by the Agencourt AMPure XP Bead Clean up kit (Beckman Coulter, Brea, California, USA) according to the manufacturer’s instructions. Samples were then subjected to multiple displacement amplification (MDA), using the Qiagen Repli-G mini kit (Qiagen, Australia) to increase the yield of bacterial DNA. All samples were quantified for *C. psittaci* genome copy number prior to and following MDA using a *C. psittaci*-specific qPCR assay targeting a 263 bp fragment of the *C. psittaci*-specific ORF_O607 gene using *C. psittaci* F3 and B3 primers^24^.

### Genome sequencing and assembly

For the Horse_pl sample, fragmentation of genomic DNA, and PCR amplification of tagged DNA were performed using the Nextera system (Illumina). Sequencing libraries were pooled, normalized using bead size selection (SPRI beads, Beckman Coulter) and quantitated on the Agilent 2100 Bioanalyzer, with High Sensitivity DNA kit. Paired-end 250 nt reads were generated using MiSeq V2 chemistry on an Illumina Miseq at the University of Technology Sydney. De novo genome assembly was carried out using SPAdes v3.9^35^ using the—careful flag to reduce potential misassembly events. Maxbin v2.2.1^36^ was used to cluster contigs according to abundance and GC content, thereby separating chlamydial and non-chlamydial contigs. Finally, CheckM v1.0.6^37^ was used to assess the quality of the clustering process.

For the remaining samples, following Illumina gDNA shotgun library preparation with bead size selection, whole-genome sequencing was carried out on an Illumina HiSeq 2500 platform, generating 125 bp paired-end reads at the Australian Genome Research Facility (AGRF), Parkville, Australia. Read quality for each sample was assessed with FastQC v.0.11.2, prior to trimming, read mapping, and de novo assembly using CLC Genomics workbench (Qiagen, USA). Initial read mapping to the reference *C. psittaci* 6BC, as well as Horse_pl chromosomes and plasmids was performed in CLC Genomics to determine the read depth for each sample and the length of genome covered. De novo assembled contigs for each sample were analysed in discontiguous BLAST to identify chlamydial contigs and contaminating DNA. Contig ordering and manual curation was conducted using Geneious mapper with high sensitivity and up to five times iteration in Geneious 10 workbench^38^, following automated annotation of draft genomes in RAST^39^. Genomes are deposited in Genbank under CP025423-CP025424 for Horse_Pl chromosome and plasmid, and PJPX00000000 for 10652_placenta; PJPY00000000 for 9945_foetus, PJPZ00000000 for 8882_foetus and PJQA00000000 for 8882_placenta. The single-217 kbp contigs for samples 9945_placenta and CR394 were also deposited in Genbank under MG823182 AND MG823181, respectively.

### Phylogenetic and comparative genomic analyses

To assess phylogenetic relationships between the newly sequenced *C. psittaci* strains from the epizootic cases, and to other previously described Australian and global *C. psittaci* strains, the core 271 kbp genome was extracted from the whole-genome MAFFT alignment of 18 publicly available genomes of *C. psittaci* from a variety of hosts (6BC (NC_017287), M56 (CP0037950, WS_RT (NC_018622), GR9(GD) (NC_018620), Mat116 (CP002744), 99DC5 (KE356190), NJ1 (NC_018626), CP3 (NC_018625), 01DC12 (NC_019391), MN (NC_018627), VS225 (NC_018621), WC (NC_018624), 84_55 (CP003790), C19/98 (NZ_KE356193), 08DC60 (NC_017290), Fr Da (NZ_LZSA00000000), CR009 (NZ_LZRX00000000), Po An (NZ_LZRG00000000), Zo Pa (NZ_LZRY00000000), HoRe_upper (NZ_LZRE00000000)), generated in Geneious v10^38^. This core genome contig was also used to re-map the reads of the poorly assembled genomes (with <20× average coverage) from this study. A mid-point rooted RaXML phylogenetic tree with 500 bootstraps and GTR CAT I model was constructed using multiple sequence MAFFT alignment of the 271 kbp core genome of 27 *C. psittaci* strains, as implemented in Geneious v10.

Out of a total of seven samples, five equine *C. psittaci* genomes sequenced in this study assembled with sufficient read depth and almost 100% genome length to enable further fine-detailed comparative genomic analyses.

### Molecular epidemiology of the equine epizootic *C. psittaci*strains

Multilocus sequence typing analysis (MLST) was successfully applied on 22 *C. psittaci-*positive DNA samples according to the scheme developed by Pannekoek et al.^29, 40^, targeting the partial fragments of seven conserved chlamydial housekeeping (HK) genes. Typed *C. psittaci-*positive DNA were extracted from placental (*n* = 10) and foetal (*n* = 9) swabs, a foetal tissue (*n* = 1), a pooled foetal/placental swab (*n* = 1), and a vaginal mare swab (*n* = 1) from 17 animals. Sequence type (ST) assignment for the 22 *C. psittaci* strains from this epizootic were determined and deposited at http://pubmlst.org/chlamydiales (Table S3). Using the alignment of the concatenated MLST fragments for the 22 *C. psittaci* strains described in this study and additional 22 previously described *C. psittaci* strains, we constructed a mid-point rooted Bayesian phylogenetic tree. The tree was constructed with MRBAYES^41^ with the GTR + I model, and run parameters including four Markov Chain Monte Carlo (MCMC) chains with a million generations, sampled every 1000 generations and with the first 10,000 trees were discarded as burn-in, as implemented in Geneious 10.

## Electronic supplementary material

Figure S1 Geographical location and distribution of the cases from the epizootic from this study

Table S1. qPCR results for paired foetal and placental tissues examined in this study

Table S2. Mean, median and range in chlamydial loads in placental vs foetal tissues as determined by qPCR

Table S3. MLST profiles
