# Supplementary figures and images for: An epizootic of Chlamydia psittaci equine reproductive loss associated with suspected spillover from native Australian parrots
Source: Emerg Microbes Infect. 2018 May 16;7:88. doi: 10.1038/s41426-018-0089-y (PMC5953950; doi:10.1038/s41426-018-0089-y)

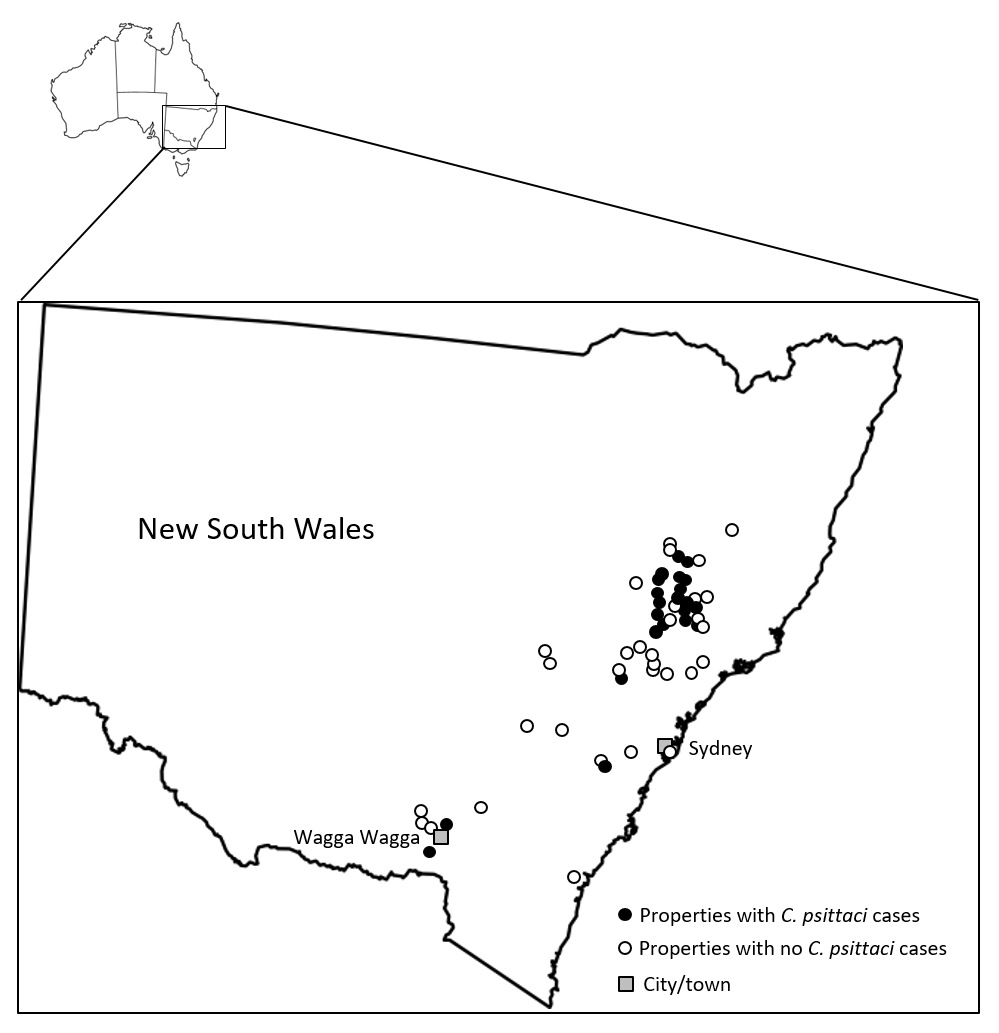

Supplement: Supplementary file 1 — Figure S1 Geographical location and distribution of the cases from the epizootic from this study [file 41426_2018_89_MOESM1_ESM.tif]
