# Supplementary material for: An epizootic of Chlamydia psittaci equine reproductive loss associated with suspected spillover from native Australian parrots
Source: Emerg Microbes Infect. 2018 May 16;7:88. doi: 10.1038/s41426-018-0089-y (PMC5953950; doi:10.1038/s41426-018-0089-y)
Supplement: Supplementary file 3 — Table S2. Mean, median and range in chlamydial loads in placental vs foetal tissues as determined by qPCR [file 41426_2018_89_MOESM3_ESM.docx]

Table S2. Mean, median and range in chlamydial loads in placental vs foetal tissues as determined by qPCR.

|  | **Placental tissue (n = 23)** | **Foetal tissue (n = 23)** |
| --- | --- | --- |
| Average chlamydial load  (gene copies/µL extract) | 2.90 × 10^5^ | 2.06 × 10^6^ |
| Median chlamydial load  (gene copies/µL extract) | 1.07 × 10^5^ | 8.31 × 10^2^ |
| Range  (gene copies/µL extract) | 3.10 × 10^3^ – 2.04 × 10^6^ | 1.5 × 10^1^ – 2.83 × 10^6^ |
